# Transitional care needs and social factors in post diagnostic support in dementia: A mixed methods study

**DOI:** 10.1002/alz70858_102692

**Published:** 2025-12-26

**Authors:** Reshma P. Mohandas, Priya Treesa Thomas, Subasree Ramakrishnan, Faheem Arshad, Suvarna Alladi

**Affiliations:** ^1^ National Institute of Mental Health and Neurosciences [NIMHANS], Bengaluru, Karnataka, India; ^2^ National Institute of Mental Health and Neurosciences, Bengaluru, Karnataka, India

## Abstract

**Background:**

Ensuring smooth care transitions of is a major challenge in healthcare settings, particularly for persons diagnosed with complex conditions like dementia and their family caregivers having diverse perspectives on the needs and service gaps in transitional care. During the transitions of care both families and persons with chronic conditions face uncertainty in each time points. This study examines transitional care needs among caregivers of persons living with dementia (PLWD) and ways to enhance the transition process and promote care continuity (Davidson et al., 2017).

**Method:**

This mixed‐methods study utilized qualitative semi‐structured interviews and quantitative assessments to explore the understanding and care needs during transitions in chronic neurological conditions. Fifty caregivers of PLWD were purposively selected. The participants were drawn from the cognitive disorders registry of the Dept of Neurology in a tertiary care neuropsychiatric centre in Bangalore India. Camberwell Needs Assessment for the Elderly (CANE), Multidimensional Scale for Perceived Social Support (MSPSS), Clinical Dementia Rating (CDR), Disability Assessment for Dementia (DAD) were administered. From this sample, twenty participants were assessed in their own homes during the home visits to explore their experiences with care transitions six months post‐discharge as part of the follow‐up plan. Thematic analysis was conducted using ATLAS.ti software, while descriptive analysis of the scales was performed using SPSS software.

**Result:**

Majority of the caregivers were spouses 30(16M, 14F) and 20(12M, 8F) were off springs. Mean age of PLWD were 62.58±9.14 and caregivers were 52.8±15.9. 24 out of 50 PLWD were having CDR score of 3 and having severe disability and majority of the duration of illness were 1 to 3 years. PLWD had significant disability as assessed by DAD (29.6±22.04). Caregivers reported moderate social support (59.8±13.79) Analysis revealed several key themes emphasizing the need for improvement in care transitions. Major themes were communication enhancement, Integrated Care Models, Patient and Family Engagement

**Conclusion:**

Extent of social support and care needs of persons with dementia varies significantly from person to person. The medical and psychosocial care support can improve the quality of care received by PLWD and their caregivers.

